# Brief report: Impact of healthcare quality on prostate specific antigen screening for the early detection of prostate cancer

**DOI:** 10.1016/j.pmedr.2019.100838

**Published:** 2019-02-25

**Authors:** Michael E. Rezaee, Charlotte E. Ward, Einar F. Sverrisson, Lawrence M. Dagrosa

**Affiliations:** a Section of Urology, Department of Surgery, Dartmouth-Hitchcock Medical Center, One Medical Center Drive, Lebanon, NH 03766, United States of America; b Center for Healthcare Studies, Feinberg School of Medicine, Northwestern University, Chicago, IL 60007, United States of America; c Center for Health Statistics, University of Chicago, Chicago, IL 60007, United States of America

**Keywords:** Prostate cancer, Cancer screening, Public health, PSA screening, Men's health, Prostate cancer, Primary care, Healthcare quality

## Abstract

With recent guidelines emphasizing patient values, patient preferences and shared decision-making in regards to prostate specific antigen (PSA) screening it is important for primary care providers and urologists to identify factors that influence men's decisions to undergo PSA screening. We sought to evaluate the impact of men's perceptions of healthcare quality on obtaining a screening PSA for the early detection of prostate cancer. A retrospective secondary data analysis was conducted of men ages 55–69 without a history of prostate cancer using 2015 Medical Expenditure Panel Survey (MEPS) data. The relationship between Consumer Assessment of Healthcare Providers and Systems (CAHPS) questions captured in MEPS and PSA screening in the last two years were assessed using multiple logistic regression. The analysis was carried out in October 2018 at Dartmouth-Hitchcock Medical Center. The final survey sample consisted of 1249 men that equated to 15,313,605.5 once weighted; 69.5% underwent PSA screening. Men who were offered help with filling out forms in the office (OR: 1.86, 95% CI: 1.14–3.01) or rated the quality of healthcare from their doctors ≥7 (OR: 1.63, 95% CI: 1.10–2.44) on a scale from 0 (worst healthcare) to 10 (best health care) had significantly greater adjusted odds of undergoing PSA screening. Men who rated the quality of healthcare delivered to them as high had significantly greater odds of undergoing PSA screening compared to those who rated it lower. Our results may suggest that improvements in healthcare quality and patient experience of care have the potential to positively influence PSA screening.

## 1 Introduction

The use of prostate specific antigen (PSA) screening for the early detection of prostate cancer remains controversial. The American Urological Association and United States Preventive Services Task Force currently recommend shared decision-making for men ages 55 to 69 who are considering PSA screening and proceeding based on a patient's values and clinical circumstances (U.S. Preventive Services Task Force, 2018; American Urological Association, 2018). In this conversation, the benefits of reducing metastatic prostate cancer diagnoses and prostate cancer-specific mortality are weighed against the potential harms of over-diagnosis and treatment (Aus et al., 2007; Loeb et al., 2014).

With increased emphasis being placed on men's values, preferences, and shared-decision making, it has become increasingly more important to understand what factors influence patient decisions to undergo PSA screening. Prior research has demonstrated a positive relationship between patient age, education, Caucasian race, income, insurance coverage, and attitudes towards physicians and PSA screening (Ogunsanya et al., 2016; Abuadas et al., 2016; Ross et al., 2009). However, limited evidence exists regarding how healthcare quality may influence men's decisions to pursue PSA screening. The purpose of this analysis was to evaluate the impact of men's perceptions of healthcare quality on PSA screening for the early detection of prostate cancer.

## 2 Methods

A retrospective secondary data analysis of 2015 Medical Expenditure Panel Survey (MEPS) data was conducted on men ages 55 to 69 years without a history of prostate cancer (Agency for Healthcare Research and Quality, 2017). As MEPS data is publicly available, institutional review board approval was not required. However, the study was conducted in accordance with the Declaration of Helsinki (World Medical Association, 2018)

PSA screening in the last two years was our primary outcome. PSA screening was determined based on a man's response to “how long since your last PSA?”, one of the preventive health questions. Our main independent variables were responses to eleven Consumer Assessment of Healthcare Providers and Systems (CAHPS) questions captured in MEPS. CAHPS questions assessed men's perceptions of healthcare quality. Men who answered, “never” or “sometimes” were considered to have disagreed with the CAHPS question; those who answered “usually” or “always” were considered to have agreed.

MEPS sampling weights, primary sampling units, and strata were used to account for the complex survey design (Agency for Healthcare Research and Quality, 2017). Corrected, weighted Pearson Chi-square and simple linear regression were used in bivariable analyses to examine the relationship between each survey item and PSA screening. Separate multivariable logistic regression models were used to evaluate the relationship between each survey item and PSA screening. We adjusted for: patient age, race, ethnicity, insurance status, educational obtainment, poverty status, self-reported health status, and marital status. Interaction terms were created for each primary survey variable and race to determine the presence of effect modification. The *p* value used for statistical significance was <0.05. The statistical software STATA 11.2 (StataCorp, College Station, TX) was used for all analyses. The analysis was carried out in October 2018 at Dartmouth-Hitchcock Medical Center.

## 3 Results

**Fig. 1:**
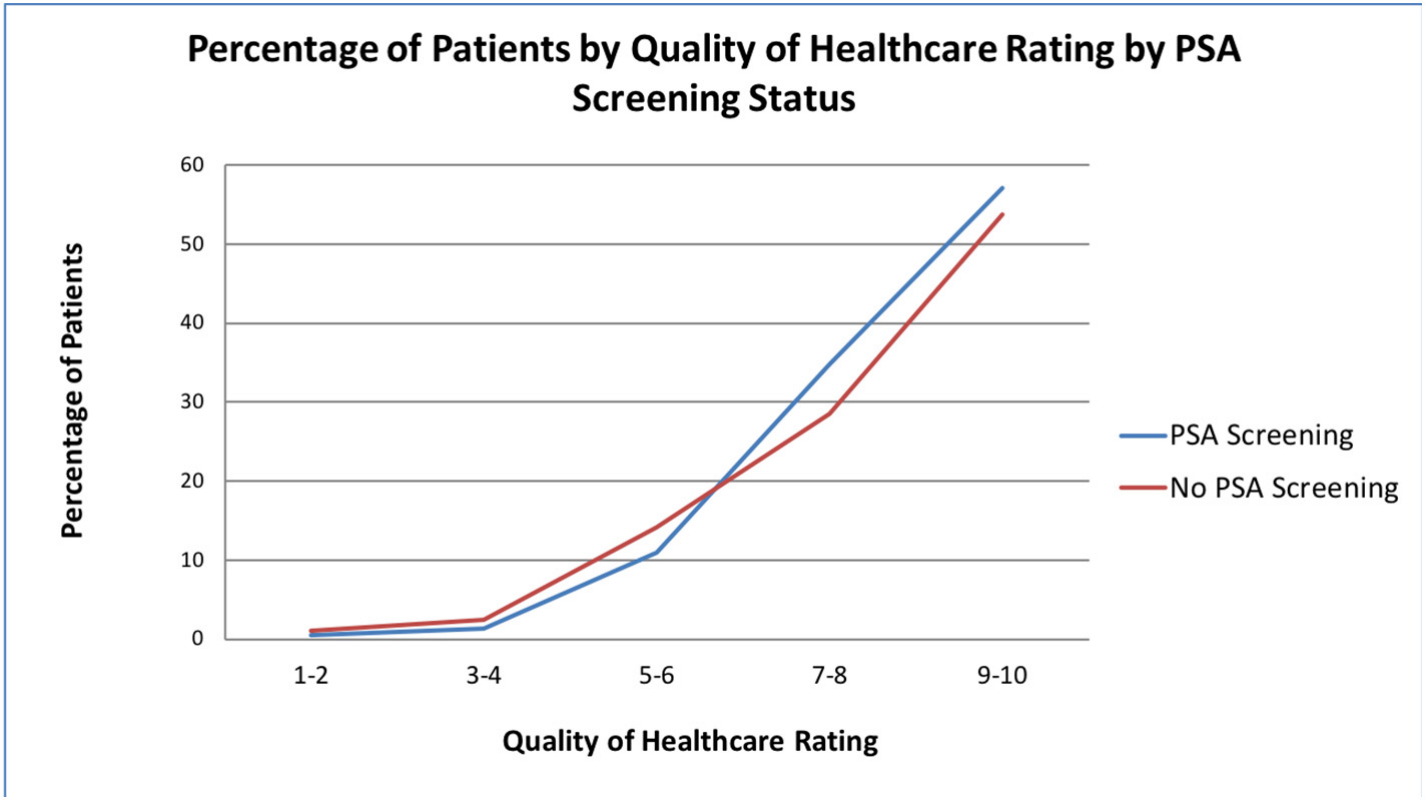
Percent of patients by quality of healthcare rating by PSA screening status.

The survey sample consisted of 1249 men that equated to 15,313,605.5 weighted individuals (see Table 1); 69.5% of these men underwent PSA screening in the last two years. Men who underwent PSA screening were more likely to be White (87.2% vs. 78.8%, *p* < 0.01), have private insurance (80.8% vs. 69.1%, *p* < 0.01), and a college degree or higher (41.0% vs. 29.6%, *p* = 0.01). In contrast, non-White men were much less likely to have undergone PSA screening in the last 2 years (12.8% vs. 21.2%, p < 0.01). A greater proportion of men underwent PSA screening if they were offered help filling out forms in a doctor's office (34.9% vs. 25.0%, *p* = 0.03). Similarly, a greater proportion of men who rated healthcare quality from their doctors ≥7 on a scale from 0 (worst healthcare possible) to 10 (best health care possible) underwent PSA screening (83.5% vs. 72.5%, p < 0.01) (see Fig. 1).

**Table 1:** Patient demographics and healthcare quality questions by PSA screening status.

	Overall 100% (15,313,605.5)	PSA screening status in the last 2 years		*P*-value
		Screening PSA 69.5% (10,642,955.8)	No screening PSA 30.5% (4,670,649.7)	
Patient Characteristics (%)				
Age (95% CI)	61.8 (61.5–62.0)	61.9 (61.6–62.3)	61.3 (60.8–61.9)	0.04
Race				
White	84.6%	87.2%	78.8%	
Black	9.0%	8.5%	10.2%	
American Indian/Alaskan	0.7%	0.3%	1.7%	
Asian/Hawaiian/Pacific	3.4%	2.3%	6.3%	
Multiracial	2.1%	1.7%	3.2%	<0.01
Non-White Race	15.4%	12.8%	21.2%	<0.01
Ethnicity - Hispanic	7.9%	7.6%	8.6%	0.47
Insurance status				
Private	77.2%	80.8%	69.1%	
Public	18.5%	16.6%	22.9%	
Uninsured	4.3%	2.6%	7.9%	<0.01
Educational obtainment				
≤ 8th Grade	3.6%	3.5%	3.9%	
9–12th Grade, no HS diploma	4.8%	4.0%	6.5%	
GED or HS Diploma	27.9%	26.3%	31.7%	
Beyond HS, Some College	25.6%	24.9%	27.2%	
4-year Bachelor Degree	20.0%	22.8%	13.9%	
Master or Doctoral Degree	17.5%	18.2%	15.7%	0.01
Poverty category				
Poor	6.6%	5.9%	8.2%	
Near poor	3.1%	2.9%	3.4%	
Low income	10.2%	10.3%	9.9%	
Middle income	21.4%	20.1%	24.3%	
High income	58.8%	60.8%	54.2%	0.19
Self-reported health status				
Poor	4.3%	4.1%	4.7%	
Fair	12.8%	11.2%	16.5%	
Good	30.5%	31.7%	27.9%	
Very Good	33.8%	33.9%	33.5%	
Excellent	18.6%	19.1%	17.4%	0.24
Marital status - not married	26.6%	25.5%	29.2%	0.21
Healthcare quality measures (Response = Usually or Always)				
Got care right away	85.3%	88.2%	79.8%	0.08
Got an appointment for health care as soon as he or she thought it was needed	87.4%	88.4%	85.2%	0.19
It was easy to get care, tests or treatment you or a doctor believed necessary	93.9%	94.5%	92.5%	0.32
Health providers listened carefully to you	93.4%	93.6%	92.8%	0.61
Health providers explained things in a way that was easy to understand	94.8%	95.3%	93.6%	0.30
Health providers showed respect for what you had to say	94.2%	95.2%	92.2%	0.09
Health providers spent enough time with you	89.4%	90.4%	86.9%	0.11
Advice given by health providers was easy to understand	96.6%	96.5%	96.8%	0.85
Health providers asked you to describe how you are going to follow their instructions	60.4%	60.7%	59.8%	0.84
Offered help with filling out forms at the office	32.1%	34.9%	25.0%	0.03
Rating of healthcare from all doctors and other health providers ≥7 from 0 (worst health care possible) to 10 (best health care possible)	80.2%	83.5%	72.5%	<0.01

In our multivariable model, men who were offered help filling out forms in the office (OR: 1.86, 95% CI: 1.14–3.01) or rated healthcare quality from their doctors ≥7 (OR: 1.63, 95% CI: 1.10–2.44) had significantly greater adjusted odds of undergoing PSA screening (see Table 2). Effect modification was observed between race and quality. Unlike non-White men, White men who were offered help filling out forms (OR: 1.94, 95% CI: 1.14–2.44) or rated healthcare quality ≥7 (OR: 1.78, 95% CI: 1.12–2.83) had significantly greater adjusted odds of undergoing screening.

**Table 2:** Crude and adjusted odds of PSA screening by quality of healthcare question.

Covariate	Crude OR (95% CI)	Adjusted OR (95% CI)
All patients		
Offered help with filling out forms at the office	1.60 (1.04–2.46)	1.86 (1.14–3.01)
Rating of healthcare from all doctors and other health providers ≥7 from 0 (worst health care possible) to 10 (best health care possible)	1.93 (1.32–2.81)	1.63 (1.10–2.44)

White patients		
Offered help with filling out forms at the office	1.79 (1.09–2.97)	1.94 (1.13–3.32)
Rating of healthcare from all doctors and other health providers ≥7 from 0 (worst health care possible) to 10 (best health care possible)	2.02 (1.33–3.07)	1.78 (1.12–2.83)

Non-White patients		
Offered help with filling out forms at the office	1.05 (0.49–2.22)	1.15 (0.42–3.17)
Rating of healthcare from all doctors and other health providers ≥7 from 0 (worst health care possible) to 10 (best health care possible)	1.32 (0.74–2.34)	1.53 (0.83–2.83)

## 4 Discussion

We found that men 55 to 69 years without a history of prostate cancer who rated the quality of care delivered to them by their doctors ≥7 (on a scale 0–10) and who received help filling out forms in the office had 63% and 86% increased odds of undergoing PSA screening, respectively. This relationship was primarily noted among White men who were found to have 78% and 94% increased odds of PSA screening, respectively. To our knowledge, we are one of the first to thoroughly investigate the relationship between perceptions of healthcare quality and PSA screening using a large, nationally representative survey.

Perceptions of healthcare quality can influence patients' willingness to undergo routine preventive health exams and screenings. Crus-Castillo et al. found that one of the most important drivers of screening mammography for breast cancer was women's perceptions of the quality of care delivered by local health centers (Cruz-Castillo et al., 2015). Chawla et al. found that patients who reported higher quality healthcare services were more likely to undergo screening colonoscopy for colon cancer (Chawla et al., 2018). In terms of PSA screening, Finney Rutten et al. observed that men who reported that providers involved them in medical decision-making had significantly greater odds of undergoing PSA screening (Finney Rutten et al., 2005). Similar to these studies, we observed a positive relationship between perceived healthcare quality and PSA screening.

Negative perceptions of healthcare quality may not only serve as barriers to accessing and engaging with healthcare, but may also hinder thoughtful and collaborative discussions between men and physicians regarding the risks/benefits of PSA screening. The most severe implication of this is the possibility of delaying or forgoing PSA screening, which may subsequently increase a patient's risk of metastatic prostate cancer and prostate cancer-specific mortality. Thus, it's important for physicians and healthcare facilities to optimize healthcare delivery and continually work to improve healthcare quality in an effort to foster and promote screening discussions, which may prevent delayed screening.

Interestingly, we observed a significant relationship between healthcare quality and PSA screening in White men, primarily. It's unclear why quality would positively impact PSA screening in White men and not in non-Whites. Unlike White men, non-White men are known to experience lower healthcare quality and health outcomes (Finney Rutten et al., 2005). It's possible that unmeasured factors in our study, such as provider-patient race concordance, communication barriers, and healthcare access were more important drivers of PSA screening in non-Whites (Finney Rutten et al., 2005; Collins et al., 2002; Saha et al., 1999). However, further research is needed to understand this disparity.

The primary strength of our study is the use of a large, nationally representative survey. In terms of limitations, MEPS variables of interest were self-reported by respondents. The validity of self-reported data can be lessened by improper recall of events and subjectivity. Additionally, MEPS is limited in its granularity to provide information on the etiology of these perceptions, which would be beneficial in determining how to improve quality. Further, although MEPS is a structured survey with standardized questions that can allow for comparisons between different groups, it can be difficult to interpret these data in clinical context. However, although we cannot express the clinical difference between a healthcare quality score of 7 compared to 6 as a cutoff, it is important to highlight that an overall relationship remains between an improved rated quality of healthcare and receipt of PSA screening. It's also difficult to assess for responder bias, as patients who chose not to participate in the survey may have a negative bias towards healthcare quality.

## 5 Conclusion

Men who rated the quality of healthcare delivered to them as high had significantly greater odds of undergoing PSA screening compared to those who rated it lower. This relationship is particularly observed in White men. Our results may suggest that improvements in healthcare quality and patient experience of care have the potential to positively influence PSA screening. However, further research is warranted to understand how objective measures of healthcare quality impact PSA screening, especially in non-White populations.

## Source of funding

None.

## Conflict of interest and disclosure statement

The authors of this manuscript have no conflicts of interest of financial disclosures to report.
